# Evaluation of the Formation of Six Beta-Carboline Alkaloids, a Class of Natural Toxins, in Meat Products Using Liquid Chromatography Tandem Mass Spectrometry

**DOI:** 10.3390/toxins17060266

**Published:** 2025-05-27

**Authors:** Kyung-Jik Lim, Do-Kyeong Lee, Han-Seung Shin

**Affiliations:** Department of Food Science and Biotechnology, Dongguk University-Seoul, 32, Dongguk-ro, Ilsandong-gu, Goyang-si 10326, Republic of Korea; kyung9209@naver.com (K.-J.L.); dodo7549@naver.com (D.-K.L.)

**Keywords:** natural toxins, beta-carboline alkaloids, livestock products, seafood, cooking method

## Abstract

Beta-carboline alkaloids (βC-alkaloids) are natural toxins found in various foods, and can also form during the thermal processing of protein-rich ingredients. This study investigated the formation of six βC-alkaloids in pork belly, beef sirloin, mackerel, and cutlassfish subjected to pan-frying, boiling, steaming, and air-frying at 170–250 °C for 2–24 min. Microwave pretreatment (1–5 min) was applied prior to cooking to assess its mitigation potential. Quantification was performed using liquid chromatography tandem mass spectrometry (LC-MS/MS). Pan-frying significantly promoted βC-alkaloid formation, with harman and norharman levels reaching up to 534.63 µg/kg and 217.06 µg/kg in beef sirloin, and 212.44 µg/kg and 533.01 µg/kg in cutlassfish, respectively. Air-frying generated lower alkaloid levels overall compared to pan-frying. Microwave pretreatment effectively mitigated alkaloid formation. The pretreatment of beef sirloin for 2 min resulted in a reduction in the norharman and harmaline levels by 78.4% and 96.5%, respectively. This study provides a comprehensive comparison of six βC-alkaloids across various food types and cooking methods, demonstrating the influence of cooking parameters on alkaloid formation. This study underscores the importance of understanding the thermal formation of natural toxins in foods and offers insight into practical strategies to minimize their occurrence in daily diets.

## 1. Introduction

Beta-carboline alkaloids (βC-alkaloids) are a diverse group of naturally occurring compounds characterized by a common indole ring structure, in which a benzene ring is fused to a pyrrole ring [1]. Examples include norharman, harman, harmine, harmol, harmalol, and harmaline. Although these molecules exert various pharmacological activities, they are simultaneously regarded as natural food-borne toxins due to both their intrinsic occurrence in plants and their thermal formation during the cooking of protein-rich foods [2,3,4,5].

βC-alkaloids are found in several botanical families (e.g., *Peganum*, *Passiflora*, *Banisteriopsis*, and *Tribulus*) and in commonly consumed commodities such as coffee, cacao, tobacco, and citrus peels [6]. During high-temperature cooking, sugars, amino acids, and indole derivatives participate in Maillard-type and Strecker pathways that generate βC-alkaloids, heterocyclic aromatic amines, and other contaminants [5,6]. While meat products have been widely investigated, βC formation in seafood remains comparatively underexplored, despite seafood’s distinct protein and lipid matrices that may affect precursor availability and reaction kinetics [7,8].

Six βC-alkaloids, including norharman, harman, harmine, harmaline, harmol, and harmalol, were selected in the present work because (i) norharman and harman are the most abundant and are classified as possible mutagens/carcinogens, showing positive responses in Ames and micronucleus assays and neuroexcitatory effects in vivo [6,9]; (ii) harmine and harmaline possess MAO-A inhibitory and hallucinogenic properties, with reported neurotoxicity and locomotor disturbances at high doses [3,4]; and (iii) harmol and harmalol are major phase I metabolites that intercalate DNA [5,7]. Considerable research interest has been directed toward the formation of harman and norharman during cooking, due to their potential roles as mutagenic and carcinogenic agents [10,11,12]. However, harmine, harmaline, harmol, and harmalol have received limited attention.

Although no official toxicological thresholds (e.g., ADI and NOAEL) have been established for these compounds, several in vivo studies suggest that β-carbolines exert biological effects at relatively high doses. For instance, renal toxicity was observed in rats fed harman at dietary levels of 1000 ppm (~50 mg/kg bw/day), but not at 500 ppm [13]. Harmine and harmaline have been shown to induce tremors and neurotoxicity at doses ranging from 10 to 20 mg/kg bw in rodents [14]. Meanwhile, harmalol demonstrated embryotoxicity in rats at a dose of 10 mg/kg [15]. A recent survey of 304 food samples confirmed the widespread presence of βC-alkaloids in over 85% of samples, yet concluded that dietary exposure was insufficient to elicit adverse effects under normal consumption [16].

The amounts of norharman and harman in meats increase sharply between 100 and 200 °C, and longer cooking times further elevate their concentrations [9,11]. By contrast, cooking techniques that employ lower temperatures or indirect heat—boiling, steaming, or air-frying—can limit Maillard progression and suppress βC formation. Microwave pretreatment has also been shown to lower the formation of thermal food mutagens in subsequent high-temperature frying [17].

Acute poisoning cases caused by the excessive ingestion of *Peganum harmala* seed extract underscore the toxicological significance of βC-alkaloids [4]. Although these compounds are not currently regulated by international agencies, their documented mutagenic and neuroactive properties—coupled with their frequent occurrence in various foods—represent an emerging toxicological concern. In contrast to heterocyclic amines, which are classified as possible human carcinogens (IARC Group 2B) and typically occur at ng/g levels, βC-alkaloids can reach concentrations of several hundred µg/kg in common dietary sources, yet they remain largely unmonitored. This disparity highlights the urgent need for comprehensive risk assessments and effective mitigation strategies.

In this study, we propose that the protein–lipid matrices of seafood generate βC-alkaloid profiles distinct from those observed in red and white meats. We further posit that brief microwave pretreatment followed by pan-frying and lower-temperature cooking will significantly reduce the overall βC burden across all sample types. To verify these propositions, we quantified six target βC-alkaloids in pork belly, beef sirloin, mackerel, and cutlassfish prepared via various cooking methods using a validated LC-MS/MS protocol. The study explores the matrix-dependent formation of βC-alkaloids, hypothesizing that seafood may exhibit distinct alkaloid profiles to pork and beef. In addition, the combined application of microwave pretreatment and the boiling and steaming cooking methods is investigated as a promising strategy to reduce overall βC exposure. These findings are expected to provide an evidence-based foundation for selecting safer cooking parameters

## 2. Results

### 2.1. Effects of Cooking Time and Temperature on Six βC-Alkaloids in Pan-Fried Meats and Seafood

**Figure 1:**
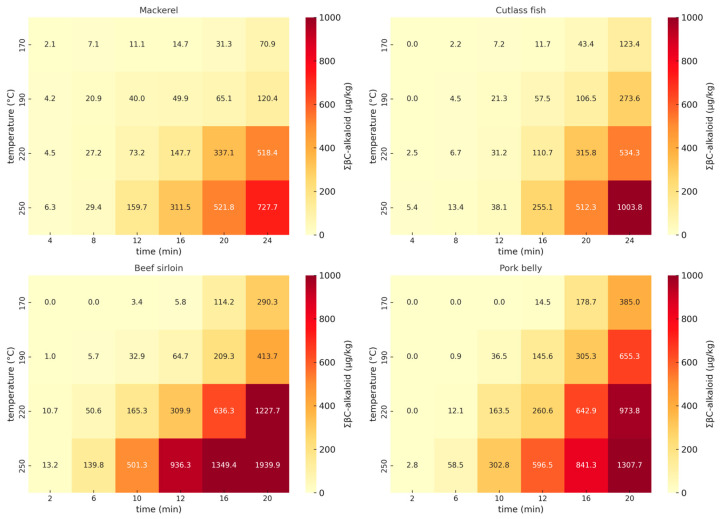
Heatmaps of total βC-alkaloid (ΣβC-alkaloid, µg/kg) levels in fish and meat samples under various heating conditions.

LC-MS/MS analysis was conducted to investigate the influence of the cooking parameters—specifically meat/seafood type, temperature, and cooking time—on the levels of the six βC-alkaloids in pan-fried samples. As illustrated in Figure 1, the formation of total ∑ βC-alkaloids varied significantly depending on both the cooking temperature and time across the four food samples. At lower temperatures (170–190 °C), the alkaloid levels remained relatively low up to 12 min. In contrast, a sharp increase was observed at higher temperatures (220–250 °C) after 8 min, particularly in beef sirloin and pork belly. These results suggest that prolonged high-temperature exposure substantially accelerates βC-alkaloid accumulation, especially in protein- and fat-rich foods

Table 1, Table 2, Table 3 and Table 4 present the concentrations of individual βC-alkaloids detected in pan-fried seafood and meat samples, including mackerel, cutlassfish, pork belly, and beef sirloin. In mackerel, norharman (2.1–174.2 μg/kg) and harman (1.7–234.0 μg/kg) were identified as the predominant βC-alkaloids, with harmaline (up to 171.2 μg/kg) and harmol (up to 49.6 μg/kg) also detected at appreciable levels, particularly under prolonged heating. Cutlassfish exhibited the highest norharman concentration among all samples in this study (up to 533.0 μg/kg), along with considerable levels of harman (up to 212.4 μg/kg) and harmaline (up to 147.6 μg/kg), especially following extended high-temperature treatments. Beef sirloin contained the greatest harmaline concentration observed (up to 886.3 μg/kg), with harman also peaking at 534.6 μg/kg, indicating this matrix’s pronounced response to thermal conditions. In pork belly, harman (up to 502.7 μg/kg) and harmaline (up to 335.1 μg/kg) were the most abundant, while other alkaloids—namely harmine, harmol, and harmalol—were detected at moderate concentrations, depending on the cooking duration.

Overall, harman and norharman consistently emerged as the most abundant βC-alkaloids, with their accumulation strongly influenced by both the temperature and heating time, suggesting a cumulative formation mechanism under prolonged thermal conditions.

Beef sirloin generally exhibited the highest overall levels of βC-alkaloids, particularly harman, harmalol, harmine, and harmaline. While norharman concentrations were most elevated in cutlassfish, harmol was most abundant in pork belly. In all food types, βC-alkaloid levels tended to increase with increasing cooking temperatures, although the extent of increase varied depending on the compound and the matrix. In beef sirloin and pork belly, the increase in the harman and harmaline concentrations was particularly pronounced, suggesting that the relatively higher fat and protein contents in these meats may enhance βC-alkaloid formation under high-temperature conditions. Notably, the harman levels in beef sirloin sharply increased at the highest temperature tested. Although the seafood samples, mackerel and cutlassfish, also showed increased βC-alkaloid concentrations with temperature, the patterns were relatively more variable. In cutlassfish, while the norharman levels increased markedly, harmol and harmalol exhibited a more moderate increase. In mackerel, the harman levels increased sharply with temperature, whereas harmine showed only a gradual increase.

### 2.2. Effects of Different Cooking Methods on the Formation of βC-Alkaloids in Meat and Seafood Samples

To evaluate the impact of various cooking methods on the formation of βC-alkaloids, four thermal processing techniques—pan-frying, boiling, steaming, and air-frying—were applied to pork belly, beef sirloin, mackerel, and cutlassfish. Each method was conducted under standardized conditions, and the levels of six individual βC-alkaloids were quantified.

**Figure 2:**
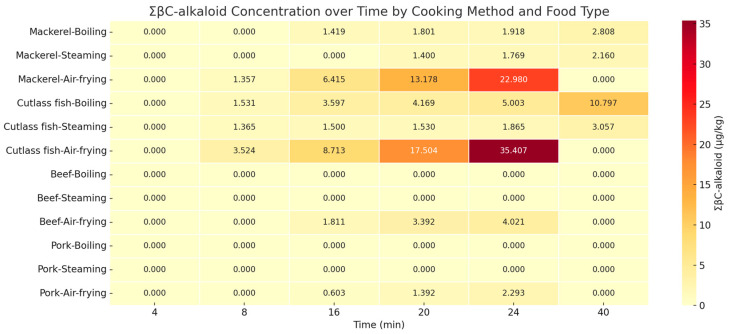
Heatmap of total βC-alkaloid (ΣβC-alkaloid, µg/kg) levels in fish and meat samples according to cooking methods and time.

The heatmap presented in Figure 2, based on the total βC-alkaloid (ΣβC-alkaloid) levels, clearly illustrates the distinct accumulation patterns over time depending on the cooking method. Under air-frying conditions (180 °C), all food types—mackerel, cutlassfish, beef, and pork—exhibited a gradual increase in βC-alkaloid concentrations over time, with mackerel (22.98 μg/kg) and cutlassfish (35.41 μg/kg) showing the highest values at 24 min. In contrast, boiling and steaming led to only slight increases, and in the case of beef and pork, βC-alkaloids were either not detected or present only at trace levels.

Pan-frying yielded the highest βC-alkaloid concentrations across all samples, with harman and norharman being the most abundant compounds. As shown in Table 1, pan-fried mackerel contained norharman and harman at 174.191 and 233.994 μg/kg, respectively. In contrast, air-frying, as presented in Table 5, resulted in norharman first being detected at 6 min (0.765 μg/kg), increasing to 22.980 μg/kg by 20 min. During the same period, harman increased from 1.297 to 10.430 μg/kg. Harmol (1.876 μg/kg), harmalol (0.961 μg/kg), and harmine (0.514 μg/kg) were detected only at later time points, while harmaline remained undetected throughout.

As shown in Table 6, cutlassfish exhibited a similar trend under air-frying, with norharman increasing from 1.568 to 35.407 μg/kg and harman reaching 6.423 μg/kg by the final time point. Other alkaloids, including harmine, harmaline, harmol, and harmalol, were detected only at 24 min, indicating delayed formation kinetics. According to Table 7 and Table 8, both beef and pork showed modest increases in norharman and harman under air-frying, with norharman ranging from 1.147 to 2.685 μg/kg and harman from 0.664 to 1.336 μg/kg. Harmaline, harmine, harmol, and harmalol were not detected throughout the heating process. A similar trend was observed in the pork samples. In all food types, boiling and steaming consistently resulted in relatively low or undetectable levels of βC-alkaloids.

As shown in Figure 2, the maximum ΣβC-alkaloid levels under boiling and steaming conditions remained low. For mackerel, the peak concentrations were 2.808 μg/kg for boiling and 2.160 μg/kg for steaming. In cutlassfish, the highest values were 10.797 μg/kg (boiling) and 3.057 μg/kg (steaming).

### 2.3. Effects of Microwave Pretreatment Duration on βC-Alkaloid Levels in Meat and Seafood Samples

The impact of microwave pretreatment duration on the formation of βC-alkaloids was evaluated in pork belly, beef sirloin, mackerel, and cutlassfish. Microwave pretreatment influenced the formation of β-carboline alkaloids across different food samples, with the extent and timing of reduction varying by sample type. As shown in Table 9, which presents the quantified concentrations of six βC-alkaloids across pretreatment durations, clear differences in the mitigation patterns were observed among mackerel, cutlassfish, beef sirloin, and pork belly. In mackerel, norharman and harman were most effectively reduced at 4 min, decreasing from 29.75 to 13.41 μg/kg and from 15.90 to 4.16 μg/kg, respectively. However, extending pretreatment to 6 min led to an increase in both compounds (norharman: 23.72 μg/kg; harman: 7.04 μg/kg), indicating a decrease in mitigation efficiency. Minor alkaloids such as harmol, harmalol, harmine, and harmaline showed similar trends, with the lowest levels generally observed between 4 and 5 min, followed by a rise at 6 min. In cutlassfish, the most notable reductions also occurred around 3–4 min (norharman: 4.04 μg/kg; harman: 0.60 μg/kg), but slight increases were observed at 5 min. Harmol and harmalol were not detected after 2 min, and harmine and harmaline also reached their lowest levels between 2 and 4 min. In contrast, beef sirloin showed the highest βC-alkaloid reduction at 2 min (norharman: 12.88 μg/kg; harman: 11.87 μg/kg), but from 3 min onward, formation increased again, with norharman reaching 97.45 μg/kg and harman 84.90 μg/kg at 5 min. Other alkaloids, including harmol, harmalol, harmine, and harmaline, were similarly reduced by over 90% at 2–3 min, but then increased notably with a longer exposure duration. Pork belly followed a comparable pattern, with the greatest reduction in norharman and harman observed at 3 min (24.98 and 15.71 μg/kg, respectively), followed by increases to 69.85 and 70.23 μg/kg at 6 min. The levels of harmol, harmalol, harmine, and harmaline were likewise minimized between 3 and 4 min, then increased again after extended pretreatment. These findings indicate that while microwave pretreatment can reduce the thermal formation of β-carboline alkaloids, an excessive exposure time compromises this effect, and the optimal reduction time varies depending on the sample matrix. Specifically, 4 min was most effective for mackerel, 3–4 min for cutlassfish, 2 min for beef sirloin, and 3 min for pork belly.

## 3. Discussion

### 3.1. Effects of Cooking Time and Temperature on the Formation of βC-Alkaloids

This study analyzed the formation of βC-alkaloids in two types of livestock products (pork belly and beef sirloin) and two types of seafood (mackerel and cutlassfish). The results revealed that both the cooking time and temperature significantly influence the formation of βC-alkaloids. These increases are associated with the intensification of the Maillard reaction, whereby tryptophan and reducing sugars react to form intermediate compounds that promote βC-alkaloid formation [18]. This aligns with earlier findings indicating that extended heat exposure facilitates the chemical transformation of amino acids like tryptophan, leading to βC-alkaloid formation [19].

β-carboline alkaloids exhibited a significant increase at temperatures above 220 °C. Notably, harmol and harmalol were not detected at lower temperatures but began to appear from 190 °C, with concentrations gradually increasing as the temperature rose. This suggests that minor βC-alkaloids may be thermally generated under specific high-temperature conditions, highlighting the need for the further elucidation of their formation mechanisms. According to previous studies, indole-containing compounds are known to form when tryptophan undergoes thermal degradation to tryptamine, which subsequently reacts with reducing sugars or aldehydes [20]. In protein-rich foods, tryptophan is likely to produce indole-based toxic substances such as βC-alkaloids under prolonged and elevated heat. These conditions may promote both Maillard and Pictet–Spengler reactions, leading to the formation of not only major compounds like norharman and harman, but also four minor βC-alkaloids [13].

Interestingly, high levels of harmine and harmaline observed at 250 °C—particularly in beef sirloin—represent a novel finding in this study and indicate the possibility of temperature-specific formation pathways based on Maillard or Pictet–Spengler-type mechanisms [21]. While previous research has reported that βC-alkaloids are generally formed under anaerobic conditions above 200 °C [22], the present study demonstrated that norharman and harman were also generated at temperatures below 200 °C. In contrast, minor βC-alkaloids were only observed at temperatures above 200 °C, implying that the tryptophan degradation products formed at these higher temperatures may serve as key intermediates in their formation.

Overall, meat products tended to generate higher levels of specific βC-alkaloids than seafood, likely due to their higher fat content and more complex protein matrices. For instance, cooked beef sirloin exhibited the highest concentration of harmaline (886.26 µg/kg), while pork belly showed elevated levels of harmol (131.548 µg/kg), suggesting that lipid-rich environments may facilitate thermally induced chemical reactions [23]. These findings align with prior reports indicating that protein–lipid interactions play a crucial role in βC-alkaloid formation [24].

In contrast to meat products, mackerel and cutlassfish exhibited distinct βC-alkaloid profiles, characterized by a predominance of norharman, while harman and harmaline were detected at relatively low concentrations. This variation likely arises from the compositional differences between seafood and terrestrial meats. One notable factor is trimethylamine-N-oxide (TMAO), a compound present in fish but not in meat, which undergoes thermal degradation to formaldehyde during cooking. Formaldehyde, in turn, can react with tryptophan or tryptamine to facilitate norharman formation [25,26]. The extended heating of seafood samples led to a marked increase in norharman levels, which may also be attributed to the high content of n-3 polyunsaturated fatty acids (PUFAs), particularly eicosapentaenoic acid (EPA) and docosahexaenoic acid (DHA), in mackerel and cutlassfish. These fatty acids are known to influence protein conformation and reactivity during thermal processing [27]. Previous studies have suggested that PUFAs modulate protein denaturation kinetics and affect the chemical environment, thereby contributing to the unique alkaloid profiles observed in seafood [28,29]. Nonetheless, the thermal formation pathways of minor βC-alkaloids remain poorly defined, necessitating further investigation into their mechanistic origins [30].

Such composition-dependent formation trends are likely to be reproduced in other animal-based foods with similar fat–protein ratios. For example, chicken (white meat) and lamb (red meat) may show comparable tendencies, and likewise, high-fat fish such as salmon and tuna could exhibit βC-alkaloid profiles similar to those observed in mackerel and cutlassfish.

### 3.2. Infulence of Different Cooking Methods on the Formation of Six βC-Alkaloids

Heterocyclic amines (HCAs), including βC-alkaloids, are generally known to form in substantial amounts during high-temperature cooking methods such as frying and grilling [31]. In this study, we observed that when thermal processing conditions—particularly temperature and time—were mitigated, the formation of these compounds signifi-cantly decreased. Boiling and steaming, which involve heat transfer via water or steam at approximately 100 °C, typically require longer cooking times compared to pan-frying [22]. Previous research has reported that such low-temperature, high-moisture cooking conditions limit the progression of the Maillard reaction, thereby reducing the for-mation of harmful compounds, including βC-alkaloids [32,33].

βC-alkaloids were not detected in pork belly or beef sirloin following boiling or steaming. However, norharman was still detected in mackerel and cutlassfish under the same conditions. This discrepancy may be attributed to endogenous metabolic processes or the unique compositional features of seafood—such as the high content of polyunsaturated fatty acids (PUFAs), including EPA and DHA—which may influence thermal reaction pathways and lead to the formation of distinct alkaloid profiles [29]. Nevertheless, the reason why such responses occur specifically in seafood remains unclear, indicating the need for further investigation.

Air-frying, a cooking technique that uses heated air to replicate the effects of traditional frying, has gained attention as a method that can reduce the formation of food-derived carcinogens compared to pan-frying or deep-frying [34,35]. This method employs indirect and gradual heating, which weakens the intensity of the Maillard reaction compare to pan-frying and consequently delays the formation of βC-alkaloids. Previous studies have shown that air-frying results in lower HCA levels in pork loin compared to high-temperature cooking methods such as deep-frying or oven cooking [33,36]. Consistent with these findings, our study also confirmed that indirect-heat-based cooking methods like air-frying may effectively suppress the formation of harmful compounds.

### 3.3. Reduction in βC-Alkaloids in Meat and Seafood via Microwave Pretreatment

Unlike conventional heating methods based on surface conduction, microwave heating involves the interaction of microwaves with polar molecules such as water, generating heat uniformly throughout the material and enabling faster and more efficient cooking [30]. Numerous studies have explored the potential of microwave pretreatment for mitigating the formation of harmful compounds in food [37]. For example, microwave pretreatment has been shown to reduce the formation of heterocyclic amines (HCAs) in fried beef patties by promoting the degradation and leaching of precursors such as creatine, amino acids, and sugars [17]. However, few studies have examined the effects of microwave pretreatment on β-carboline alkaloids. Therefore, this study aimed to evaluate the impact of microwave pretreatment on β-carboline alkaloid formation in both meat and seafood, including compounds that have not previously been studied in this context.

In beef sirloin, microwave pretreatment significantly reduced β-carboline alkaloid levels: norharman decreased by 78.4%, harmol and harmalol by 92.7% and 91.7%, respectively, and harmine and harmaline by 96.1% and 96.5%, respectively. Pork belly showed lower reductions: norharman by 59.3%, harmol and harmalol by 78.9% and 78.4%, respectively, and harmine and harmaline by 93.1% and 86.6%, respectively. In seafood, cutlassfish exhibited greater reductions than mackerel. Norharman and harman decreased by 81.9% and 85.8% in cutlassfish, respectively, compared to 54.9% and 73.8% in mackerel. Notably, harmol and harmalol were completely eliminated in cutlassfish, while reductions in mackerel were 79.2% and 82.5%, respectively. These results provide novel evidence on the formation and reduction patterns of harmol, harmalol, harmine, and harmaline, which have not been previously reported.

The optimal microwave pretreatment time to minimize β-carboline alkaloid formation varied depending on the sample: a total of 2 min for beef sirloin, 3 min for pork belly and cutlassfish, and 4 min for mackerel. These differences are likely attributable to variations in sample composition, moisture content, and physical structure [38]. Pork belly, with its higher fat and lower water contents, showed a slower reduction efficiency, whereas the high moisture content of beef sirloin facilitated the more effective leaching of precursors [39,40]. According to previous research, cutlassfish has relatively low fat and high moisture contents, whereas mackerel is considered fattier [41]. In this study, cutlassfish samples had a thinner structure than mackerel, allowing for more effective heat penetration and precursor diffusion. In contrast, the thicker tissue and uneven heating of mackerel likely limited the reduction effect, suggesting that compositional and structural factors may hinder the effectiveness of microwave pretreatment in such samples.

Microwave heating also affects macromolecules such as proteins and lipids by altering the heat distribution as the moisture levels decrease [42]. Microwaves rapidly interact with water molecules, inducing internal heating and promoting water leaching, which facilitates the removal of alkaloid precursors [43]. However, as moisture is lost, localized overheating can occur, triggering structural changes in proteins and lipids and potentially initiating the Maillard reaction. In this study, such effects were reflected in the reappearance of β-carboline alkaloids, whose levels initially declined but increased again upon prolonged heating. This reformation appears to be closely linked to moisture loss and precursor migration, which can alter molecular interactions and impact the balance between the suppression and regeneration of alkaloid compounds. These findings are consistent with previous reports supporting this mechanism [44,45]. Therefore, it is essential to optimize the microwave pretreatment conditions to minimize the formation of β-carboline alkaloids while preventing their reformation during extended thermal processing.

## 4. Conclusions

This study presents the first comprehensive quantitative analysis comparing six βC-alkaloids in pork belly, beef sirloin, mackerel, and cutlassfish, each subjected to five commonly used cooking methods. βC-alkaloids are traditionally classified as naturally occurring toxins, found in certain plant-based foods such as *Peganum* and *Passiflora* species. However, recent studies have demonstrated that they can also be formed via Maillard reactions involving amino acids, sugars, and lipids during high-temperature cooking, thereby acting as process-induced contaminants. This dual origin complicates dietary exposure assessment and underscores the importance of investigating βC-alkaloids’ thermal behavior and developing mitigation strategies from a food safety perspective.

In this study, pan-frying at temperatures above 220 °C resulted in the highest βC-alkaloid concentrations, with harmaline reaching up to 886 µg kg^−1^ in beef sirloin and norharman up to 533 µg kg^−1^ in cutlassfish. In contrast, moisture-retaining cooking methods such as boiling and steaming greatly suppressed βC-alkaloid formation, keeping most compounds below the quantification limit. Microwave pretreatment at 700 W for 2–3 min prior to pan-frying reduced the total βC-alkaloid levels by up to 90%. However, excessive pretreatment (≥5 min) caused significant moisture loss and intensified the Maillard reactions, paradoxically promoting alkaloid reformation. These findings emphasize the need for the precise control of the pretreatment parameters to maximize the reduction effect.

Distinct alkaloid formation patterns were also observed across different food matrices. Harman, harmaline, and harmol were predominantly detected in pork and beef, whereas norharman was dominant in seafood. This discrepancy may be attributed to the high content of n-3 polyunsaturated fatty acids (EPA and DHA) in seafood, which could affect the thermal reactivity of precursors. These results suggest that relatively simple adjustments—such as temperature regulation, the use of moist heat cooking methods, and brief microwave pretreatment—can effectively reduce dietary exposure to βC-alkaloids.

Future research should expand the range of tested food matrices to include not only a broader variety of marine products such as shrimp, squid, and seaweed, but also various types of protein-rich foods including red meats (e.g., lamb), white meats (e.g., chicken), and fatty fish (e.g., salmon), in order to validate the formation characteristics of βC-alkaloids across a more diverse set of dietary sources. This study confirms that both the selection of the cooking conditions and the application of microwave pretreatment are effective in reducing βC-alkaloid formation, providing a direct scientific basis for practical cooking guidelines applicable to everyday meal preparation.

## 5. Materials and Methods

### 5.1. Chemicals and Reagents

Nine βC-alkaloid standards were used in this study. Norharman (98%), harman (98%), harmine (98%), and harmaline (95%) were purchased from Sigma-Aldrich (St. Louis, MO, USA). Harmol (98%) was obtained from TCI (Tokyo, Japan), and harmalol (62%) was obtained from PhytoLab (Vestenbergsgreuth, Germany). Deuterium-labeled compounds used as internal standards (ISTDs)—norharman-d7 (97%), harman-d3 (95%), and harmine-d3 (96%)—were supplied by TRC (Toronto, ON, Canada). All solvents and reagents were of high-performance liquid chromatography (HPLC) grade, including ammonium formate (Sigma-Aldrich, Bangalore, India), formic acid (Wako, Osaka, Japan), water (Samchun, Seoul, Republic of Korea), and acetonitrile (ACN; Samchun). QuEChERS extraction salt packets (P/N 5982–7650), containing 4 g of MgSO4, 1 g of NaCl, 0.5 g of disodium citrate sesquihydrate, and 1 g of trisodium citrate, and dispersive SPE kits (P/N 5982–5156), containing 150 mg of primary secondary amine (PSA), 150 mg of end-capped C18, and 900 mg of MgSO4, were purchased from Agilent (Santa Clara, CA, USA). All meat and seafood samples were purchased from local markets in the Republic of Korea.

### 5.2. Thermal Processing Conditions Applied to Meat and Seafood Samples Using Various Cooking Methods

Four cooking methods—pan-frying, boiling, steaming, and air-frying—were applied to meat and seafood samples under controlled conditions. Pan-frying was performed at surface temperatures of 170, 190, 220, and 250 °C. Heating durations were set to 2, 4, 6, 8, 10, 12, 16, and 20 min for meat products and 4, 6, 8, 10, 12, 16, 20, and 24 min for seafood products. Boiling and steaming were conducted for the same durations as pan-frying, with an additional 40 min40-min time point included to evaluate the prolonged thermal exposure. For air-frying, the samples were preheated at 180 °C and heated for the same time durations used in pan-frying. Cooking temperatures of 170–250 °C for up to 24 min were selected on the basis of previously reported kinetic studies [46].

During thermal treatment, surface temperatures were monitored using an infrared thermometer (IT300-1, CAS, Yang-Ju Si, Republic of Korea) for pan-frying, while boiling and steaming temperatures were measured using a contact thermometer (SK-1110, Sato Keiryoki MFG, Tokyo, Japan). In air-frying, the internal temperature was maintained at 180 °C. The sample sizes were standardized as follows: mackerel (7 cm × 5 cm × 1.5 cm), cutlassfish (7 cm × 5 cm × 1.0 cm), beef sirloin (5 cm × 5 cm × 0.5 cm), and pork belly (5 cm × 5 cm × 0.5 cm). All samples were purchased before thermal treatment.

### 5.3. Thermal Treatment with Microwave Pretreatment Followed by Pan-Frying

To investigate the mitigating effect of microwave pretreatment on the formation of βC-alkaloids, the same samples used in the pan-frying experiments were subjected to microwave heating before pan-frying. Microwave pretreatment was conducted using a 700 W microwave oven (2450 MHz, KR-U209Q, Daewoo Electronics, Gun-Po si, Republic of Korea) in for 1 to 5 min. The cControl samples were pan-fried directly without microwave exposure. After pretreatment, livestock cuts were pan-fried at 220 °C for 10 min, whereas seafood filetsfillets were pan-fried at the same temperature for 12 min. The mMicrowave pretreatment conditions (700 W, 1–5 min) were adapted from Felton et al. [17].

### 5.4. Sample Preparation for the Analysis of Six βC-Alkaloids

The sample preparation method for extracting βC-alkaloids from food samples was optimized based on the QuEChERS method (European Standard EN 15662), incorporating the use of acetic acid in the extraction solvent and sodium citrate in the extraction salt [2]. First, 2 g of the homogenized sample was spiked with 10 μL of the ISTD mixture, followed by the addition of 5 mL deionized water. The concentration of each internal standard in the spiked sample was adjusted to 50 μg/kg. Next, the extraction solvent, ACN (10 mL), was added and vortexed for 1 min. Subsequently, the extraction salt mix containing 4 g of MgSO4, 1 g of NaCl, 0.5 g of disodium citrate sesquihydrate, and 1 g of trisodium citrate was added. Afterward, the extraction tube was shaken vigorously by in a multitube vortex mixer (2000 rpm for 1 min). This was followed by centrifugation at 4000 rpm for 5 min, and then 5 mL of the supernatant was transferred to a dispersive solid-phase extraction (d-SPE) tube containing 150 mg of PSA, 150 mg of end-capped C18, and 900 mg of MgSO4. The d-SPE tube was shaken at 2000 rpm for 1 min and then centrifuged at 4000 rpm for 5 min. Subsequently, the 2 mL of supernatant was transferred into another tube and evaporated under nitrogen gas at 40 °C for 20 min using a flow rate of 500 mL/min. The dried residue was reconstituted in 200 μL of 80% (*v*/*v*) ACN for LC-MS/MS analysis. The sample preparation method used in this study was adapted with minor modifications from our previously validated protocol [16].

### 5.5. Quantitative Analysis of Six βC-Alkaloids by LC-MS/MS

To analyze the six βC-alkaloids, LC-MS/MS was utilized, a method recognized for its exceptional sensitivity and precision. Chromatographic separation was performed using a SCIEX ExionLC™ AD UHPLC system (Concord, ON, Canada), equipped with a Kinetex^®^ C18 column (100 mm × 2.1 mm, 2.6 μm; Phenomenex, Torrance, CA, USA) that was maintained at a constant temperature of 40 °C for analyte separation.

Mobile phase A consisted of HPLC-grade water containing 0.1% (*v*/*v*) formic acid and 1 mM ammonium formate, while mobile phase B consisted of ACN with 0.1% (*v*/*v*) formic acid. The gradient started at 10% B, held for 0.5 min, then increased to 33% B over 2.1 min, followed by a rise to 44% B over 3.5 min. The composition was then ramped up to 95% B, held for 1.5 min, and followed by a post -run of 3.0 min to ensure adequate column re-equilibration. The flow rate and injection volume were 0.4 mL/min and 1 μL, respectively.

The tandem mass spectrometry setup allows for additional fragmentation of the molecules, generating distinct spectral data that facilitate the accurate determination of the chemical structure and characteristics of the compounds. The analysis was conducted using an AB Sciex API 3200 triple quadrupole mass spectrometer equipped with a Turbo V™ ion source operating in positive electrospray ionization (ESI) positive mode.

The curtain gas (CUR) was set to 20 (a.u.), the collision gas (CAD) to 5 (a.u.), and the ion spray voltage (IS) to 3500 V. The system temperature (TEM) was maintained at 550 °C. Ion source gas 1 (GS1) and gas 2 (GS2) were set to 50 (a.u.) each. Additionally, the interface heater was turned on to facilitate the ionization process. These conditions were optimized to ensure the effective ionization and detection of the target βC-alkaloids. Detailed information on the optimized MRM conditions is provided in Supplementary Table S1.

The analytical methods employed in this study were validated in our previous work [16]. The method was validated for linearity, sensitivity, recovery, and precision. The calibration curves were constructed over the concentration range of 0.1–100 μg/kg, and validation was performed using pork, beef, mackerel, and cutlassfish. Detailed results are provided in Supplementary Table S2. Chromatograms of the six βC-alkaloids’s standard compounds and three internal standards are shown in Supplementary Figure S1, obtained using the preparation and analytical procedures applied in this study, which —are presented in Supplementary Figure S2. Furthermore, validation was conducted using raw (uncooked) pork, beef, mackerel, and cutlassfish matrices. No βC-alkaloids were detected in these uncooked samples, indicating that the alkaloids quantified in this study were formed exclusively during thermal processing. These raw (uncooked) samples were used as controls to assess the baseline levels of βC-alkaloids prior to thermal processing.

### 5.6. Statistical Analysis

All experiments were conducted in triplicate, and the results are expressed as means ± standard deviation (SD). To analyze the data on the six βC-alkaloids in both livestock products and seafood, Tukey’s HSD test was applied in IBM SPSS Statistics (Chicago, IL, USA). This post hoc test was used to compare group means and determine significant differences, with a threshold of p < 0.05. Statistical analyses were carried out using IBM SPSS Statistics for Windows (version 27.0; IBM Corp., Armonk, NY, USA).

## Figures and Tables

**Table 1:** Contents of six βC-alkaloids in pan-fried mackerel at four different temperatures ^(1),(2),(3)^.

Temp (°C)	βC-Alkaloids (µg/kg)	Time (min)							
		4	6	8	10	12	16	20	24
170	Norharman	2.114 ± 0.207 ^A,a^	4.431 ± 0.114 ^A,b^	5.417 ± 0.103 ^A,bc^	6.304 ± 0.231 ^A,cd^	7.126 ± 0.202 ^A,d^	9.045 ± 0.165 ^A,e^	24.702 ± 0.304 ^A,f^	35.075 ± 1.350 ^A,g^
	Harman	ND	ND	1.662 ± 0.147 ^A,a^	1.817 ± 0.179 ^A,a^	2.401 ± 0.136 ^A,b^	2.858 ± 0.240 ^A,c^	3.374 ± 0.160 ^A,d^	16.224 ± 0.195 ^A,e^
	Harmol	ND	ND	ND	ND	0.798 ± 0.014 ^A,a^	1.174 ± 0.118 ^A,ab^	1.486 ± 0.026 ^A,b^	5.187 ± 0.418 ^A,c^
	Harmalol	-	ND	ND	0.363 ± 0.081 ^A,a^	0.733 ± 0.019 ^A,b^	1.004 ± 0.028 ^A,bc^	1.076 ± 0.040 ^A,c^	3.367 ± 0.297 ^A,d^
	Harmine	-	-	ND	ND	ND	0.601 ± 0.048 ^A,a^	0.703 ± 0.031 ^A,a^	3.900 ± 0.172 ^A,b^
	Harmaline	-	-	-	ND	ND	ND	ND	7.164 ± 0.285 ^A,a^
190	Norharman	2.219 ± 0.044 ^A,a^	9.593 ± 0.199 ^B,b^	14.291 ± 0.330 ^B,c^	18.276 ± 1.429 ^B,d^	21.649 ± 0.097 ^B,e^	23.599 ± 0.619 ^B,e^	30.933 ± 1.111 ^B,f^	56.195 ± 0.967 ^B,g^
	Harman	1.967 ± 0.200 ^A,a^	2.411 ± 0.140 ^A,ab^	3.900 ± 0.066 ^B,bc^	4.749 ± 0.140 ^B,c^	8.993 ± 0.118 ^B,d^	11.629 ± 0.808 ^B,e^	15.746 ± 0.746 ^B,f^	26.77 ± 1.374 ^B,g^
	Harmol	ND	ND	1.736 ± 0.149 ^A,a^	2.058 ± 0.153 ^A,ab^	2.654 ± 0.180 ^A,b^	4.493 ± 0.219 ^AB,c^	6.514 ± 0.273 ^B,d^	11.451 ± 0.771 ^B,e^
	Harmalol	ND	ND	1.016 ± 0.050 ^A,a^	1.51 ± 0.051 ^B,ab^	1.779 ± 0.071 ^B,b^	2.027 ± 0.167 ^AB,b^	3.041 ± 0.275 ^B,c^	8.700 ± 0.397 ^B,d^
	Harmine	-	-	ND	1.533 ± 0.095 ^A,a^	2.110 ± 0.144 ^A,a^	3.641 ± 0.150 ^B,b^	3.783 ± 0.226 ^B,b^	7.663 ± 0.868 ^B,c^
	Harmaline	-	-	ND	1.429 ± 0.091 ^A,a^	2.854 ± 0.114 ^A,b^	4.509 ± 0.120 ^A,c^	5.131 ± 0.171 ^A,d^	9.602 ± 0.140 ^B,e^
220	Norharman	2.858 ± 0.199 ^B,a^	10.107 ± 0.356 ^B,b^	16.342 ± 1.269 ^C,c^	24.163 ± 0.928 ^C,d^	27.845 ± 1.269 ^C,d^	47.333 ± 2.705 ^C,e^	83.021 ± 2.289 ^C,f^	122.522 ± 0.477 ^C,g^
	Harman	1.680 ± 0.184 ^A,a^	2.852 ± 0.164 ^B,a^	5.344 ± 0.140 ^C,ab^	11.819 ± 0.382 ^C,bc^	15.185 ± 0.913 ^C,c^	25.425 ± 0.589 ^C,d^	49.545 ± 3.850 ^C,e^	105.316 ± 6.421 ^C,f^
	Harmol	ND	ND	2.105 ± 0.099 ^B,a^	2.440 ± 0.209 ^A,ab^	3.201 ± 0.120 ^A,b^	5.263 ± 0.634 ^B,c^	13.594 ± 0.672 ^C,d^	20.616 ± 0.140 ^C,e^
	Harmalol	ND	0.381 ± 0.043 ^A,a^	1.356 ± 0.055 ^B,b^	3.131 ± 0.145 ^C,c^	10.806 ± 0.130 ^D,d^	21.056 ± 1.071 ^C,e^	27.078 ± 0.420 ^D,f^	40.576 ± 1.285 ^D,g^
	Harmine	-	ND	0.535 ± 0.058 ^A,ab^	2.076 ± 0.140 ^B,b^	4.760 ± 0.060 ^C,c^	10.939 ± 0.459 ^D,d^	35.489 ± 0.874 ^D,e^	58.084 ± 1.183 ^D,f^
	Harmaline	ND	ND	1.504 ± 0.242 ^A,a^	4.025 ± 0.234 ^B,a^	11.452 ± 0.682 ^B,b^	37.670 ± 2.235 ^B,c^	128.327 ± 2.445 ^C,d^	171.241 ± 4.215 ^C,e^
250	Norharman	3.971 ± 0.244 ^D,a^	11.931 ± 0.859 ^D,b^	17.889 ± 0.189 ^D,b^	28.454 ± 1.259 ^D,c^	63.971 ± 2.580 ^D,d^	90.514 ± 1.408 ^D,e^	120.478 ± 2.847 ^D,f^	174.191 ± 5.320 ^D,g^
	Harman	2.367 ± 0.130 ^B,a^	4.010 ± 0.007 ^C,a^	5.788 ± 0.064 ^D,a^	16.293 ± 0.743 ^D,b^	54.024 ± 1.571 ^D,c^	125.627 ± 2.690 ^D,d^	176.627 ± 4.875 ^D,e^	233.994 ± 1.852 ^D,f^
	Harmol	ND	ND	2.368 ± 0.069 ^D,ab^	5.736 ± 0.451 ^B,b^	14.719 ± 1.862 ^B,c^	25.718 ± 2.766 ^C,d^	33.760 ± 2.890 ^D,e^	49.633 ± 0.750 ^D,f^
	Harmalol	ND	0.381 ± 0.043 ^A,a^	1.356 ± 0.055 ^B,b^	3.131 ± 0.145 ^C,c^	10.806 ± 0.130 ^D,d^	21.056 ± 1.071 ^C,e^	27.078 ± 0.420 ^D,f^	40.576 ± 1.285 ^D,g^
	Harmine	-	ND	0.535 ± 0.058 ^A,ab^	2.076 ± 0.140 ^B,b^	4.760 ± 0.060 ^C,c^	10.939 ± 0.459 ^D,d^	35.489 ± 0.874 ^D,e^	58.084 ± 1.183 ^D,f^
	Harmaline	ND	ND	1.504 ± 0.242 ^A,a^	4.025 ± 0.234 ^B,a^	11.452 ± 0.682 ^B,b^	37.670 ± 2.235 ^B,c^	128.327 ± 2.445 ^C,d^	171.241 ± 4.215 ^C,e^

^(1)^ Data represent mean ± SD of three analyses/treatments (*n* = 3); ND, the levels were lower than the limit of quantification; and -, the levels were lower than the limit of detection. ^(2)^ Different superscript uppercase letters in columns (temperature) and lowercase letters in rows (time) indicate statistically significant differences (*p* < 0.05). ^(3)^ Post hoc comparisons were conducted using Tukey’s HSD test in SPSS to analyze the differences between groups.

**Table 2:** Contents of six βC-alkaloids in pan-fried cutlassfish at four different temperatures ^(1),(2),(3)^.

Temp (°C)	βC-Alkaloids (µg/kg)	Time (min)							
		4	6	8	10	12	16	20	24
170	Norharman	ND	2.144 ± 0.149 ^A,ab^	2.241 ± 0.075 ^A,ab^	3.510 ± 0.401 ^A,bc^	5.711 ± 0.250 ^A,c^	9.787 ± 0.367 ^A,d^	33.806 ± 1.863 ^A,e^	104.191 ± 2.764 ^A,f^
	Harman	ND	ND	ND	1.167 ± 0.014 ^A,a^	1.501 ± 0.058 ^A,ab^	1.921 ± 0.106 ^A,b^	5.886 ± 0.475 ^A,c^	10.970 ± 0.466 ^A,d^
	Harmol	ND	ND	ND	ND	ND	ND	1.535 ± 0.222 ^A,a^	2.923 ± 0.420 ^A,b^
	Harmalol	ND	ND	ND	ND	ND	ND	0.628 ± 0.058 ^A,a^	0.943 ± 0.035 ^A,b^
	Harmine	ND	ND	ND	ND	ND	ND	ND	0.965 ± 0.085 ^A,a^
	Harmaline	ND	ND	ND	ND	ND	ND	1.592 ± 0.092 ^A,a^	3.396 ± 0.214 ^A,b^
190	Norharman	ND	2.943 ± 0.156 ^B,ab^	3.255 ± 0.424 ^B,b^	4.226 ± 0.353 ^A,b^	13.71 ± 0.858 ^B,c^	37.231 ± 1.041 ^B,d^	61.077 ± 1.211 ^B,e^	145.75 ± 2.556 ^B,f^
	Harman	ND	ND	1.261 ± 0.072 ^A,ab^	1.385 ± 0.083 ^A,ab^	2.535 ± 0.042 ^B,b^	10.571 ± 0.313 ^B,c^	28.645 ± 0.659 ^B,d^	70.066 ± 1.248 ^B,e^
	Harmol	ND	ND	ND	ND	1.094 ± 0.090 ^A,a^	2.561 ± 0.100 ^A,b^	4.581 ± 0.081 ^A,c^	8.834 ± 0.144 ^B,d^
	Harmalol	ND	ND	ND	ND	0.508 ± 0.073 ^A,a^	1.553 ± 0.142 ^A,b^	2.962 ± 0.112 ^B,c^	5.113 ± 0.441 ^B,d^
	Harmine	ND	ND	ND	ND	0.473 ± 0.041 ^A,a^	0.902 ± 0.094 ^A,b^	2.357 ± 0.205 ^A,c^	12.005 ± 0.241 ^B,d^
	Harmaline	ND	ND	ND	1.417 ± 0.034 ^A,ab^	2.985 ± 0.081 ^A,bc^	4.663 ± 0.494 ^A,cd^	6.874 ± 0.437 ^B,d^	31.828 ± 2.519 ^B,e^
220	Norharman	2.518 ± 0.072 ^A,a^	3.827 ± 0.264 ^C,a^	4.971 ± 0.182 ^C,a^	6.315 ± 0.832 ^B,a^	19.686 ± 0.770 ^C,b^	63.05 ± 0.368 ^C,c^	147.296 ± 2.150 ^C,d^	268.828 ± 5.540 ^C,e^
	Harman	ND	0.958 ± 0.024 ^A,ab^	1.716 ± 0.060 ^A,ab^	2.347 ± 0.123 ^B,ab^	3.685 ± 0.337 ^C,b^	22.055 ± 0.200 ^C,c^	88.663 ± 1.756 ^C,d^	132.718 ± 2.516 ^C,e^
	Harmol	ND	ND	ND	ND	1.258 ± 0.112 ^A,a^	5.843 ± 0.424 ^B,b^	16.246 ± 2.058 ^B,c^	27.803 ± 1.072 ^C,d^
	Harmalol	ND	ND	ND	ND	0.688 ± 0.074 ^A,a^	2.552 ± 0.404 ^B,b^	4.917 ± 0.469 ^C,c^	10.641 ± 0.707 ^C,d^
	Harmine	ND	ND	ND	ND	0.766 ± 0.111 ^B,a^	1.266 ± 0.070 ^B,a^	9.898 ± 0.374 ^B,b^	17.307 ± 0.412 ^C,c^
	Harmaline	ND	ND	ND	1.983 ± 0.067 ^B,ab^	5.087 ± 0.339 ^B,b^	15.888 ± 1.430 ^B,c^	48.828 ± 2.076 ^C,d^	76.969 ± 3.285 ^C,e^
250	Norharman	4.410 ± 0.257 ^B,a^	5.314 ± 0.345 ^D,a^	9.204 ± 0.534 ^D,ab^	14.905 ± 0.368 ^C,bc^	20.171 ± 0.488 ^C,c^	164.718 ± 2.072 ^D,d^	285.481 ± 3.060 ^D,e^	533.008 ± 5.122 ^D,f^
	Harman	0.962 ± 0.067 ^A,a^	1.016 ± 0.094 ^A,a^	3.113 ± 0.356 ^B,a^	3.353 ± 0.230 ^c,ab^	7.190 ± 0.336 ^D,b^	41.992 ± 1.765 ^D,c^	121.814 ± 1.784 ^D,d^	212.438 ± 2.867 ^D,e^
	Harmol	ND	ND	1.076 ± 0.100 ^A,a^	1.484 ± 0.124 ^A,a^	1.850 ± 0.118 ^B,a^	12.827 ± 1.204 ^C,b^	23.835 ± 1.169 ^C,c^	58.255 ± 1.179 ^D,d^
	Harmalol	ND	ND	ND	ND	1.191 ± 0.093 ^B,a^	4.965 ± 0.567 ^C,b^	10.216 ± 0.757 ^D,c^	18.619 ± 1.082 ^D,d^
	Harmine	ND	ND	ND	ND	0.834 ± 0.028 ^B,a^	3.089 ± 0.054 ^C,b^	12.645 ± 0.074 ^C,c^	33.796 ± 0.716 ^D,d^
	Harmaline	ND	ND	ND	2.115 ± 0.104 ^B,ab^	6.819 ± 0.259 ^C,b^	27.506 ± 2.182 ^C,c^	58.311 ± 2.969 ^D,d^	147.642 ± 4.124 ^D,e^

^(1)^ Data represent mean ± SD of three analyses/treatments (*n* = 3); ND, the levels were lower than the limit of quantification. ^(2)^ Different superscript uppercase letters in columns (temperature) and lowercase letters in rows (time) indicate statistically significant differences (*p* < 0.05). ^(3)^ Post hoc comparisons were conducted using Tukey’s HSD test in SPSS to analyze the differences between groups.

**Table 3:** Contents of six βC-alkaloids in pan-fried beef sirloin at four different temperatures ^(1),(2),(3)^.

Temp (°C)	βC-Alkloids (µg/kg)	Time (min)							
		2	4	6	8	10	12	16	20
170	Norharman	ND	ND	ND	1.413 ± 0.025 ^A,a^	1.567 ± 0.020 ^A,a^	2.894 ± 0.062 ^A,b^	36.472 ± 0.278 ^A,c^	62.819 ± 0.253 ^A,d^
	Harman	ND	ND	ND	1.153 ± 0.016 ^A,ab^	1.796 ± 0.038 ^A,bc^	2.943 ± 0.046 ^A,c^	40.342 ± 0.837 ^A,d^	108.955 ± 1.066 ^A,e^
	Harmol	-	-	ND	ND	ND	ND	8.725 ± 0.355 ^A,a^	41.698 ± 1.338 ^A,b^
	Harmalol	-	-	-	-	ND	ND	8.859 ± 0.178 ^A,a^	26.217 ± 0.439 ^A,b^
	Harmine	-	-	-	-	-	ND	3.777 ± 0.121 ^A,a^	16.736 ± 0.880 ^A,b^
	Harmaline	-	-	-	-	ND	ND	16.005 ± 0.461 ^A,a^	33.916 ± 0.334 ^A,b^
190	Norharman	ND	1.070 ± 0.015 ^A,ab^	1.686 ± 0.049 ^A,ab^	2.651 ± 0.010 ^B,b^	12.420 ± 0.292 ^B,c^	21.876 ± 0.337 ^B,d^	62.965 ± 1.588 ^B,e^	84.801 ± 1.330 ^B,f^
	Harman	1.001 ± 0.004 ^A,a^	2.197 ± 0.031 ^A,ab^	4.009 ± 0.090 ^A,bc^	4.629 ± 0.040 ^B,c^	13.491 ± 0.176 ^B,d^	24.369 ± 0.384 ^B,e^	75.105 ± 0.911 ^B,f^	135.765 ± 1.869 ^B,g^
	Harmol	-	ND	ND	ND	2.649 ± 0.053 ^A,a^	8.036 ± 0.178 ^A,b^	14.416 ± 0.312 ^B,c^	53.502 ± 1.298 ^B,d^
	Harmalol	-	-	ND	0.325 ± 0.029 ^A,a^	1.308 ± 0.046 ^A,b^	3.061 ± 0.118 ^A,c^	20.030 ± 0.445 ^B,d^	50.402 ± 0.466 ^B,e^
	Harmine	-	-	-	ND	0.915 ± 0.025 ^A,a^	2.108 ± 0.041 ^A,b^	4.140 ± 0.006 ^B,c^	19.872 ± 0.764 ^B,d^
	Harmaline	-	-	ND	ND	2.117 ± 0.035 ^A,a^	5.212 ± 0.142 ^A,b^	32.659 ± 0.505 ^B,c^	69.336 ± 1.006 ^B,d^
220	Norharman	3.138 ± 0.259 ^A,a^	6.385 ± 0.127 ^B,b^	17.700 ± 0.438 ^B,c^	22.092 ± 0.652 ^C,d^	56.987 ± 1.131 ^C,e^	87.854 ± 0.799 ^C,f^	163.199 ± 0.690 ^C,f^	197.632 ± 1.536 ^C,g^
	Harman	4.380 ± 0.291 ^B,a^	5.565 ± 0.051 ^B,a^	16.354 ± 0.624 ^B,b^	22.812 ± 0.992 ^C,c^	57.700 ± 1.178 ^C,d^	102.869 ± 1.291 ^C,e^	245.632 ± 0.783 ^C,f^	441.524 ± 1.632 ^C,g^
	Harmol	0.847 ± 0.026 ^A,a^	1.500 ± 0.051 ^A,a^	5.244 ± 0.145 ^A,b^	9.865 ± 0.041 ^A,c^	15.505 ± 0.279 ^B,d^	32.998 ± 0.708 ^B,e^	58.110 ± 0.592 ^C,f^	79.494 ± 1.221 ^C,g^
	Harmalol	0.495 ± 0.012 ^A,a^	1.195 ± 0.012 ^A,a^	3.056 ± 0.062 ^A,b^	5.775 ± 0.169 ^B,c^	9.908 ± 0.350 ^B,d^	27.816 ± 0.360 ^B,e^	47.483 ± 0.819 ^C,f^	60.263 ± 0.637 ^C,g^
	Harmine	0.459 ± 0.029 ^A,a^	0.614 ± 0.015 ^A,a^	1.486 ± 0.033 ^A,a^	2.775 ± 0.109 ^A,b^	5.359 ± 0.077 ^B,c^	10.815 ± 0.246 ^B,d^	16.573 ± 0.606 ^C,e^	53.757 ± 0.801 ^C,f^
	Harmaline	1.392 ± 0.013 ^A,a^	1.843 ± 0.049 ^A,a^	6.786 ± 0.136 ^A,b^	10.37 ± 0.150 ^A,c^	19.861 ± 0.224 ^B,d^	47.519 ± 0.588 ^B,e^	105.267 ± 1.185 ^C,f^	395.026 ± 1.558 ^C,g^
250	Norharman	4.553 ± 0.104 ^B,a^	21.342 ± 1.236 ^c,b^	40.122 ± 0.963 ^C,c^	52.635 ± 0.390 ^D,d^	113.850 ± 1.176 ^D,e^	166.368 ± 0.531 ^D,f^	184.383 ± 1.647 ^D,g^	217.055 ± 1.833 ^D,h^
	Harman	4.047 ± 0.104 ^B,a^	16.981 ± 0.541 ^C,b^	44.294 ± 1.668 ^C,c^	66.087 ± 0.554 ^D,d^	218.813 ± 0.579 ^D,e^	349.656 ± 0.925 ^D,f^	454.306 ± 1.747 ^D,g^	534.629 ± 2.444 ^D,h^
	Harmol	1.060 ± 0.079 ^B,a^	7.471 ± 0.409 ^B,b^	14.26 ± 0.352 ^B,c^	19.292 ± 0.304 ^B,d^	35.243 ± 0.408 ^C,e^	43.164 ± 0.566 ^C,f^	72.951 ± 1.155 ^D,g^	113.104 ± 1.591 ^D,h^
	Harmalol	1.401 ± 0.068 ^B,a^	10.217 ± 0.261 ^B,b^	12.997 ± 0.524 ^B,c^	21.994 ± 0.558 ^C,d^	26.409 ± 0.756 ^C,e^	34.484 ± 0.461 ^C,f^	54.199 ± 0.711 ^D,g^	75.612 ± 0.863 ^D,h^
	Harmine	0.538 ± 0.015 ^B,a^	2.103 ± 0.031 ^B,b^	7.810 ± 0.037 ^B,c^	9.280 ± 0.088 ^B,d^	17.635 ± 0.343 ^C,e^	43.271 ± 0.417 ^C,f^	66.184 ± 0.519 ^D,g^	113.229 ± 0.900 ^D,h^
	Harmaline	1.576 ± 0.093 ^B,a^	7.605 ± 0.279 ^B,b^	20.296 ± 0.181 ^B,c^	45.554 ± 0.327 ^B,d^	89.375 ± 1.438 ^C,e^	299.406 ± 1.440 ^C,f^	517.417 ± 1.485 ^D,g^	886.260 ± 1.488 ^D,h^

^(1)^ Data represent mean ± SD of three analyses/treatments (*n* = 3); ND, the levels were lower than the limit of quantification; and -, the levels were lower than the limit of detection. ^(2)^ Different superscript uppercase letters in columns (temperature) and lowercase letters in rows (time) indicate statistically significant differences (*p* < 0.05). ^(3)^ Post hoc comparisons were conducted using Tukey’s HSD test in SPSS to analyze the differences between groups.

**Table 4:** Contents of six βC-alkaloids in pan-fried pork belly at four different temperatures ^(1),(2),(3)^.

Temp (°C)	βC-Alkloids (µg/kg)	Time (min)							
		2	4	6	8	10	12	16	20
170	Norharman	ND	ND	ND	ND	ND	3.683 ± 0.287 ^A,a^	44.343 ± 0.630 ^A,b^	91.228 ± 1.205 ^A,c^
	Harman	ND	ND	ND	ND	ND	2.116 ± 0.104 ^A,a^	65.905 ± 0.583 ^A,b^	160.948 ± 1.141 ^A,c^
	Harmol	-	-	-	-	ND	0.930 ± 0.007 ^A,a^	21.752 ± 0.637 ^A,b^	28.018 ± 0.538 ^A,c^
	Harmalol	-	-	-	-	-	ND	8.406 ± 0.193 ^A,a^	10.818 ± 0.361 ^A,b^
	Harmine	-	-	-	-	ND	5.965 ± 0.450 ^A,a^	16.437 ± 0.319 ^A,b^	
	Harmaline	-	-	-	-	ND	1.841 ± 0.162 ^A,a^	21.877 ± 0.188 ^A,b^	93.981 ± 1.815 ^A,c^
190	Norharman	ND	ND	ND	3.164 ± 0.072 ^A,a^	12.857 ± 0.101 ^A,b^	54.423 ± 0.723 ^B,c^	79.478 ± 0.706 ^B,d^	107.496 ± 0.596 ^B,e^
	Harman	ND	ND	0.932 ± 0.043 ^A,a^	2.933 ± 0.230 ^A,b^	14.974 ± 0.270 ^A,c^	59.796 ± 1.000 ^B,d^	148.833 ± 1.048 ^B,e^	303.693 ± 1.040 ^B,f^
	Harmol	-	ND	ND	ND	3.269 ± 0.042 ^A,a^	13.051 ± 0.154 ^B,b^	26.183 ± 0.725 ^B,c^	57.929 ± 0.509 ^B,d^
	Harmalol	-	-	-	-	2.130 ± 0.067 ^A,a^	6.589 ± 0.010 ^A,b^	11.964 ± 0.101 ^B,c^	25.000 ± 0.435 ^B,d^
	Harmine	-	-	-	ND	ND	3.104 ± 0.114 ^A,a^	12.552 ± 0.524 ^B,b^	25.606 ± 0.387 ^B,c^
	Harmaline	-	-	-	1.579 ± 0.206 ^A,a^	3.298 ± 0.334 ^A,b^	8.622 ± 0.140 ^B,c^	26.333 ± 0.314 ^B,d^	135.59 ± 1.157 ^B,e^
220	Norharman	ND	0.820 ± 0.017 ^A,a^	5.281 ± 0.144 ^A,b^	37.719 ± 0.753 ^B,c^	60.438 ± 0.565 ^B,d^	79.735 ± 0.677 ^C,e^	133.857 ± 1.407 ^C,f^	169.837 ± 1.781 ^C,g^
	Harman	ND	ND	3.714 ± 0.037 ^B,a^	28.730 ± 0.475 ^B,b^	59.749 ± 1.364 ^B,c^	98.009 ± 0.623 ^C,d^	278.264 ± 1.828 ^C,e^	404.763 ± 1.448 ^C,f^
	Harmol	ND	ND	1.745 ± 0.037 ^A,a^	8.244 ± 0.180 ^A,b^	12.485 ± 0.648 ^B,c^	22.793 ± 0.978 ^C,d^	52.898 ± 0.893 ^C,e^	77.422 ± 1.462 ^C,f^
	Harmalol	-	-	ND	4.769 ± 0.248 ^A,a^	6.465 ± 0.437 ^B,b^	11.801 ± 0.387 ^B,c^	28.095 ± 0.794 ^C,d^	51.353 ± 1.150 ^C,e^
	Harmine	-	-	ND	2.034 ± 0.052 ^A,a^	5.924 ± 0.252 ^A,b^	12.650 ± 0.169 ^B,c^	25.051 ± 0.804 ^C,d^	38.405 ± 1.114 ^C,e^
	Harmaline	-	-	1.357 ± 0.122 ^A,a^	13.59 ± 0.494 ^B,b^	18.409 ± 0.853 ^B,c^	35.603 ± 0.860 ^C,d^	124.777 ± 0.817 ^C,e^	232.057 ± 1.302 ^C,f^
250	Norharman	1.025 ± 0.034 ^A,a^	13.810 ± 0.314 ^B,b^	23.782 ± 1.017 ^B,c^	57.838 ± 0.841 ^C,d^	83.563 ± 0.943 ^C,e^	125.03 ± 0.772 ^D,f^	167.82 ± 1.663 ^D,g^	195.105 ± 1.061 ^D,h^
	Harman	1.766 ± 0.226 ^A,a^	16.623 ± 0.475 ^A,b^	19.619 ± 0.896 ^C,b^	77.722 ± 1.213 ^C,c^	131.238 ± 1.439 ^C,d^	283.772 ± 1.411 ^D,e^	338.825 ± 2.871 ^D,f^	502.671 ± 2.972 ^D,g^
	Harmol	ND	2.663 ± 0.217 ^A,a^	5.195 ± 0.201 ^B,b^	19.825 ± 1.230 ^B,c^	28.291 ± 0.503 ^C,d^	39.756 ± 0.646 ^D,e^	60.901 ± 0.661 ^D,f^	131.548 ± 1.390 ^D,g^
	Harmalol	-	1.115 ± 0.016 ^A,a^	2.615 ± 0.025 ^A,a^	8.619 ± 0.072 ^B,b^	15.303 ± 0.422 ^C,c^	27.433 ± 1.393 ^C,d^	39.788 ± 1.491 ^D,e^	64.380 ± 1.674 ^D,f^
	Harmine	-	1.049 ± 0.030 ^A,a^	1.624 ± 0.171 ^A,a^	3.681 ± 0.183 ^B,b^	7.051 ± 0.300 ^B,c^	19.656 ± 0.620 ^C,d^	33.349 ± 0.540 ^D,e^	78.894 ± 1.706 ^D,f^
	Harmaline	ND	3.899 ± 0.061 ^A,a^	5.664 ± 0.127 ^B,b^	29.485 ± 0.842 ^C,c^	37.345 ± 1.063 ^C,d^	100.892 ± 0.705 ^D,e^	200.614 ± 1.531 ^D,f^	335.061 ± 2.449 ^D,g^

^(1)^ Data represent mean ± SD of three analyses/treatments (*n* = 3); ND, the levels were lower than the limit of quantification; and -, the levels were lower than the limit of detection. ^(2)^ Different superscript uppercase letters in columns (temperature) and lowercase letters in rows (time) indicate statistically significant differences (*p* < 0.05). ^(3)^ Post hoc comparisons were conducted using Tukey’s HSD test in SPSS to analyze the differences between groups.

**Table 5:** Contents of six βC-alkaloids in mackerel cooked using three different methods ^(1),(2),(3)^.

Cooking Method	βC-Alkaloids (µg/kg)	Time (min)								
		4	6	8	10	12	16	20	24	40
Boiling	Norharman	ND	ND	ND	0.960 ± 0.096 ^a^	1.124 ± 0.194 ^ab^	1.419 ± 0.150 ^b^	1.801 ± 0.144 ^c^	1.918 ± 0.110 ^c^	2.808 ± 0.123 ^d^
	Harman	ND	ND	ND	ND	ND	ND	ND	ND	ND
	Harmol	ND	ND	ND	ND	ND	ND	ND	ND	ND
	Harmalol	-	-	-	-	-	-	-	-	-
	Harmine	-	-	-	-	-	-	-	-	-
	Harmaline	-	-	-	-	-	-	-	-	-
Steaming	Norharman	ND	ND	ND	ND	ND	ND	1.400 ± 0.049 ^a^	1.769 ± 0.097 ^b^	2.160 ± 0.063 ^c^
	Harman	ND	ND	ND	ND	ND	ND	ND	ND	ND
	Harmol	ND	ND	ND	ND	ND	ND	ND	ND	ND
	Harmalol	-	-	-	-	-	-	-	-	-
	Harmine	-	-	-	-	-	-	-	-	-
	Harmaline	-	-	-	-	-	-	-	-	-
Air- frying (180 °C)	Norharman	ND	0.765 ± 0.106 ^ab^	1.357 ± 0.136 ^b^	1.511 ± 0.184 ^b^	4.921 ± 0.290 ^c^	6.415 ± 0.116 ^d^	13.178 ± 0.642 ^e^	22.980 ± 1.728 ^f^	
	Harman	ND	ND	ND	ND	1.297 ± 0.126 ^a^	2.471 ± 0.059 ^b^	5.406 ± 0.509 ^c^	10.430 ± 0.202 ^d^	
	Harmol	ND	ND	ND	ND	ND	ND	0.854 ± 0.029 ^a^	1.876 ± 0.120 ^b^	
	Harmalol	-	-	-	-	-	ND	0.629 ± 0.017 ^a^	0.961 ± 0.118 ^b^	
	Harmine	-	-	-	-	ND	ND	0.324 ± 0.019 ^a^	0.514 ± 0.019 ^b^	
	Harmaline	-	-	-	-	-	-	ND	ND	

^(1)^ Data represent mean ± SD of three analyses/treatments (*n* = 3); ND, the levels were lower than the limit of quantification; and -, the levels were lower than the limit of detection. ^(2)^ Different letters in each row indicate statistically significant differences among time points (*p* < 0.05). ^(3)^ Post hoc comparisons were conducted using Tukey’s HSD test in SPSS to analyze the differences between groups.

**Table 6:** Contents of six βC-alkaloids in cutlassfish cooked using three different methods ^(1),(2),(3)^.

Cooking Method	βC-Alkaloids (µg/kg)	Time (min)								
		4	6	8	10	12	16	20	24	40
Boiling	Norharman	ND	1.441 ± 0.252 ^a^	1.531 ± 0.066 ^a^	2.379 ± 0.098 ^b^	2.555 ± 0.178 ^b^	3.597 ± 0.132 ^c^	4.169 ± 0.258 ^c^	5.003 ± 0.270 ^d^	10.797 ± 0.616 ^e^
	Harman	ND	ND	ND	ND	ND	ND	ND	ND	ND
	Harmol	-	ND	ND	ND	ND	ND	ND	ND	ND
	Harmalol	-	-	-	-	-	-	-	-	-
	Harmine	-	-	-	-	-	-	-	-	-
	Harmaline	-	-	-	-	-	-	-	-	-
Steaming	Norharman	ND	1.300 ± 0.064 ^ab^	1.365 ± 0.061 ^ab^	1.385 ± 0.141 ^ab^	1.453 ± 0.117 ^ab^	1.500 ± 0.102 ^abc^	1.530 ± 0.077 ^bc^	1.865 ± 0.093 ^b^	3.057 ± 0.220 ^d^
	Harman	ND	ND	ND	ND	ND	ND	ND	ND	ND
	Harmol	ND	ND	ND	ND	ND	ND	ND	ND	ND
	Harmalol	-	-	-	-	-	-	-	-	-
	Harmine	-	-	-	-	-	-	-	-	-
	Harmaline	-	-	-	-	-	-	-	-	-
Air- frying (180 °C)	Norharman	ND	1.568 ± 0.194 ^ab^	3.524 ± 0.326 ^bc^	4.3 ± 0.084 ^bc^	5.058 ± 0.329 ^c^	8.713 ± 0.250 ^d^	17.504 ± 1.244 ^e^	35.407 ± 2.648 ^f^	
	Harman	ND	ND	ND	ND	ND	ND	ND	6.423 ± 0.229 ^a^	
	Harmol	ND	ND	ND	ND	ND	ND	ND	2.879 ± 0.331 ^a^	
	Harmalol	-	-	-	-	-	-	-	0.542 ± 0.133 ^a^	
	Harmine	-	-	-	-	-	-	-	0.673 ± 0.029 ^a^	
	Harmaline	-	-	-	-	-	-	-	1.896 ± 0.160 ^a^	

^(1)^ Data represent mean ± SD of three analyses/treatments (*n* = 3); ND, the levels were lower than the limit of quantification; and -, the levels were lower than the limit of detection. ^(2)^ Different letters in each row indicate statistically significant differences among time points (*p* < 0.05). ^(3)^ Post hoc comparisons were conducted using Tukey’s HSD test in SPSS to analyze the differences between groups.

**Table 7:** Contents of six βC-alkaloids in beef sirloin cooked using three different methods ^(1),(2),(3)^.

Cooking Method	βC-Alkaloids (µg/kg)	Time (min)								
		4	6	8	10	12	16	20	24	40
Boiling	Norharman	-	-	-	-	-	-	-	-	-
	Harman	-	-	-	-	-	-	-	-	-
	Harmol	-	-	-	-	-	-	-	-	-
	Harmalol	-	-	-	-	-	-	-	-	-
	Harmine	-	-	-	-	-	-	-	-	-
	Harmaline	-	-	-	-	-	-	-	-	-
Steam	Norharman	-	-	-	-	-	-	-	-	-
	Harman	-	-	-	-	-	-	-	-	-
	Harmol	-	-	-	-	-	-	-	-	-
	Harmalol	-	-	-	-	-	-	-	-	-
	Harmine	-	-	-	-	-	-	-	-	-
	Harmaline	-	-	-	-	-	-	-	-	-
Air- frying (180 °C)	Norharman	-	-	-	-	ND	1.147 ± 0.057 ^a^	2.208 ± 0.262 ^b^	2.685 ± 0.049 ^c^	
	Harman	-	-	-	-	ND	0.664 ± 0.091 ^a^	1.184 ± 0.093 ^b^	1.336 ± 0.097 ^c^	
	Harmol	-	-	-	-	-	ND	ND	ND	
	Harmalol	-	-	-	-	-	ND	ND	ND	
	Harmine	-	-	-	-	-	-	-	-	
	Harmaline	-	-	-	-	-	-	-	-	

^(1)^ Data represent mean ± SD of three analyses/treatments (*n* = 3); ND, the levels were lower than the limit of quantification; and -, the levels were lower than the limit of detection. ^(2)^ Different letters in each row indicate statistically significant differences among time points (*p* < 0.05). ^(3)^ Post hoc comparisons were conducted using Tukey’s HSD test in SPSS to analyze the differences between groups.

**Table 8:** Contents of six βC-alkaloids in pork belly cooked using three different methods ^(1),(2),(3)^.

Cooking Method	βC-Alkaloids (µg/kg)	Time (min)								
		4	6	8	10	12	16	20	24	40
Boiling	Norharman	-	-	-	-	-	-	-	-	-
	Harman	-	-	-	-	-	-	-	-	-
	Harmol	-	-	-	-	-	-	-	-	-
	Harmalol	-	-	-	-	-	-	-	-	-
	Harmine	-	-	-	-	-	-	-	-	-
	Harmaline	-	-	-	-	-	-	-	-	-
Steaming	Norharman	-	-	-	-	-	-	-	-	-
	Harman	-	-	-	-	-	-	-	-	-
	Harmol	-	-	-	-	-	-	-	-	-
	Harmalol	-	-	-	-	-	-	-	-	-
	Harmine	-	-	-	-	-	-	-	-	-
	Harmaline	-	-	-	-	-	-	-	-	-
Air- frying (180 °C)	Norharman	-	-	-	-	ND	0.603 ± 0.178 ^a^	1.392 ± 0.236 ^b^	1.541 ± 0.192 ^b^	
	Harman	-	-	-	-	-	ND	ND	0.752 ± 0.106 ^a^	
	Harmol	ND	ND	ND	ND	ND	ND	ND	ND	
	Harmalol	-	-	-	-	-	-	-	-	
	Harmine	-	-	-	-	-	-	-	-	
	Harmaline	-	-	-	-	-	-	-	ND	

^(1)^ Data represent mean ± SD of three analyses/treatments (*n* = 3); ND, the levels were lower than the limit of quantification; and -, the levels were lower than the limit of detection. ^(2)^ Different letters in each row indicate statistically significant differences among time points (*p* < 0.05). ^(3)^ Post hoc comparisons were conducted using Tukey’s HSD test in SPSS to analyze the differences between groups.

**Table 9:** Effect of microwave pretreatment duration on βC-alkaloid levels in four types of meat ^(1),(2),(3)^.

Food Sample	βC-Alkaloids (µg/kg)	Time (min)					
		Con.	1	2	3	4	5
Pork belly	Norharman	61.432 ± 1.483 ^e^	49.530 ± 1.193 ^d^	42.482 ± 0.871 ^c^	24.983 ± 0.677 ^a^	30.765 ± 1.008 ^b^	69.850 ± 1.521 ^f^
	Harman	60.876 ± 0.634 ^e^	55.219 ± 0.286 ^d^	48.371 ± 0.342 ^c^	15.707 ± 0.211 ^a^	19.819 ± 0.621 ^b^	70.234 ± 0.968 ^f^
	Harmol	12.897 ± 0.454 ^e^	6.568 ± 0.351 ^c^	5.250 ± 0.215 ^b^	2.721 ± 0.137 ^a^	6.023 ± 0.286 ^bc^	8.473 ± 0.308 ^d^
	Harmalol	6.749 ± 0.309 ^e^	2.568 ± 0.221 ^c^	1.858 ± 0.027 ^ab^	1.456 ± 0.047 ^a^	2.255 ± 0.110 ^bc^	4.177 ± 0.162 ^d^
	Harmine	6.593 ± 0.326 ^f^	3.260 ± 0.041 ^d^	2.534 ± 0.138 ^c^	0.454 ± 0.035 ^a^	1.126 ± 0.172 ^b^	3.727 ± 0.060 ^e^
	Harmaline	18.171 ± 0.375 ^e^	10.177 ± 0.398 ^d^	8.99 ± 0.185 ^c^	2.439 ± 0.103 ^a^	4.145 ± 0.083 ^b^	17.921 ± 0.481 ^e^
Beef sirloin	Norharman	59.516 ± 0.887 ^e^	38.805 ± 1.273 ^d^	12.882 ± 0.480 ^a^	15.859 ± 0.407 ^b^	34.935 ± 1.141 ^c^	97.448 ± 0.800 ^f^
	Harman	60.131 ± 0.557 ^d^	29.458 ± 0.205 ^c^	11.865 ± 0.506 ^a^	13.838 ± 0.150 ^b^	29.653 ± 1.150 ^c^	84.902 ± 0.832 ^e^
	Harmol	16.067 ± 0.234 ^c^	6.665 ± 0.136 ^b^	1.178 ± 0.018 ^a^	2.097 ± 0.222 ^a^	6.209 ± 0.189 ^b^	15.099 ± 3.204 ^c^
	Harmalol	11.146 ± 0.307 ^e^	5.540 ± 0.174 ^c^	0.922 ± 0.060 ^a^	1.373 ± 0.151 ^a^	2.910 ± 0.164 ^b^	7.335 ± 0.110 ^d^
	Harmine	6.422 ± 0.225 ^d^	2.866 ± 0.221 ^b^	0.253 ± 0.019 ^a^	0.391 ± 0.038 ^a^	0.648 ± 0.070 ^a^	4.548 ± 0.166 ^c^
	Harmaline	20.348 ± 0.321 ^d^	4.056 ± 0.243 ^b^	0.715 ± 0.053 ^a^	0.884 ± 0.023 ^a^	1.528 ± 0.036 ^a^	15.070 ± 0.802 ^c^
Mackerel	Norharman	29.749 ± 0.533 ^f^	26.591 ± 0.152 ^e^	19.664 ± 0.298 ^c^	17.531 ± 0.155 ^b^	13.414 ± 0.183 ^a^	23.722 ± 0.306 ^d^
	Harman	15.899 ± 0.096 ^f^	13.652 ± 0.150 ^e^	6.399 ± 0.093 ^c^	5.059 ± 0.021 ^b^	4.164 ± 0.055 ^a^	7.040 ± 0.167 ^d^
	Harmol	3.976 ± 0.057 ^e^	2.533 ± 0.107 ^d^	1.702 ± 0.049 ^c^	1.029 ± 0.056 ^b^	0.828 ± 0.047 ^a^	1.580 ± 0.048 ^c^
	Harmalol	2.436 ± 0.029 ^e^	1.864 ± 0.034 ^d^	0.665 ± 0.026 ^c^	0.553 ± 0.025 ^b^	0.427 ± 0.009 ^a^	0.698 ± 0.023 ^c^
	Harmine	3.069 ± 0.204 ^d^	1.986 ± 0.085 ^c^	0.351 ± 0.037 ^a^	0.238 ± 0.058 ^a^	0.404 ± 0.171 ^a^	0.915 ± 0.089 ^b^
	Harmaline	3.368 ± 0.088 ^e^	2.800 ± 0.033 ^d^	0.809 ± 0.042 ^c^	0.577 ± 0.044 ^b^	0.314 ± 0.027 ^a^	0.930 ± 0.012 ^c^
Cutlassfish	Norharman	22.294 ± 0.436 ^e^	10.105 ± 0.317 ^d^	5.909 ± 0.098 ^b^	4.040 ± 0.127 ^a^	4.683 ± 0.141 ^a^	6.813 ± 0.197 ^c^
	Harman	4.241 ± 0.111 ^e^	1.839 ± 0.081 ^d^	0.905 ± 0.035 ^b^	0.601 ± 0.033 ^a^	0.785 ± 0.034 ^b^	1.126 ± 0.057 ^c^
	Harmol	1.379 ± 0.033 ^b^	1.111 ± 0.064 ^a^	ND	-	ND	ND
	Harmalol	0.821 ± 0.060 ^c^	0.354 ± 0.016 ^a^	ND	-	ND	0.442 ± 0.019 ^b^
	Harmine	0.998 ± 0.019 ^e^	0.549 ± 0.007 ^c^	0.41 ± 0.008 ^b^	ND	0.268 ± 0.008 ^a^	0.636 ± 0.009 ^d^
	Harmaline	5.766 ± 0.142 ^d^	3.686 ± 0.152 ^c^	2.731 ± 0.136 ^b^	1.228 ± 0.035 ^a^	1.397 ± 0.036 ^a^	2.678 ± 0.109 ^b^

^(1)^ Data represent mean ± SD of three analyses/treatments (*n* = 3); ND, the levels were lower than the limit of quantification; and -, the levels were lower than the limit of detection. ^(2)^ Different letters in each row indicate statistically significant differences among time points (*p* < 0.05). ^(3)^ Post hoc comparisons were conducted using Tukey’s HSD test in SPSS to analyze the differences between groups.

## Data Availability

The original contributions presented in this study are included in the article. Further inquiries can be directed to the corresponding author(s).

## Supplementary Materials

The following supporting information can be downloaded at https://www.mdpi.com/article/10.3390/toxins17060266/s1, Table S1. Optimized MRM conditions for βC-alkaloid detection. Table S2. Validation data for the βC alkaloids in four kinds of food sample. Figure S1. LC-MS/MS chromatograms of β-carboline standards and internal standards (50 μg/kg) using selected precursor–product ion transitions. Figure S2. LC-MS/MS chromatograms of pork belly spiked with β-carboline standards and internal standard (50 μg/kg).
