# Supplementary material for: Evaluation of the Formation of Six Beta-Carboline Alkaloids, a Class of Natural Toxins, in Meat Products Using Liquid Chromatography Tandem Mass Spectrometry
Source: Toxins (Basel). 2025 May 27;17(6):266. doi: 10.3390/toxins17060266 (PMC12197532; doi:10.3390/toxins17060266)
Supplement: Supplementary file 1 [file toxins-17-00266-s001.zip › toxins-3613685-supplementary.pdf]

Evaluation of the Formation of Six β-Carboline Alkaloids, a Class of Natural Toxins, in Meat Products Using LC-MS/MS

Kyung-Jik Lim, Do-Kyeong Lee, and Han-Seung Shin*

Supplementary Table S1**.** Optimized MRM conditions for βC-alkaloid detection.

Compound	Precursor ion (*m*/*z*)	Product ion (*m*/*z*)	DP (V)	EP (V)	CEP (V)	CE (V)	CXP (V)	Realtive ion ratio
Norharman	169.0	169.0	70	10	12	13	2.3	0.15
		142.0	70	10	12	37	2.3	
		115.0*	70	10	12	48	2.3	
Harman	183.0	183.0	55	10	12	11	2.0	0.11
		168.0	55	10	12	40	4.0	
		115.0*	55	10	12	45	3.0	
Harmine	199.0	199.0	65	10	13	13	3.0	0.80
		171.0	65	10	13	34	3.0	
		103.0*	65	10	13	44	2.5	
Harmalol	201.0	201.0	50	10	11	10	3.0	0.11
		185.0	50	10	11	45	3.0	
		160.0*	50	10	11	28	3.0	
Harmol	213.0	213.0	60	10	15	13	3.0	0.79
		198.0	60	10	15	34	4.0	
		170.0*	60	10	15	44	4.0	
Harmaline	215.0	215.0	55	10	12	13	5.0	0.88
		172.0*	55	10	12	40	4.0	
		131.0	55	10	12	52	4.0	
Norharman-d_7**	176.0	176.0	65	10	10	10	2.0	0.16
		148.0	65	10	10	37	2.5	
		120.0*	65	10	10	50	3.0	
Harman-d_3**	186.0	186.0	60	10	11	12	4.0	0.12
		168.0	60	10	11	40	2.6	
		115.0*	60	10	11	45	2.7	
Harmine-d_3**	216.0	216.0	55	10	12	10	2.0	0.82
		170.0*	55	10	12	44	2.2	
		169.0	55	10	12	57	2.1	

*: Product ion used for quantification.

**: Internal standard.

Supplementary Table S2. Validation data for the βC alkaloids in four kinds of food sample

Food sample	Compound	Linearity(R^2)	LOD (μg/kg)	LOQ (μg/kg)	Intra-day (n=3)		Inter-day (n=3)	
					Recovery(%)	Precision(%RSD)	Recovery(%)	Precision(%RSD)
Mackerel	Norharman	0.999	0.090	0.273	98.9 ± 3.2	3.17 ± 0.51	99.9 ± 2.5	3.01 ± 0.46
	Harman	0.999	0.197	0.596	98.4 ± 1.9	3.14 ± 0.40	86.7 ± 2.4	3.01 ± 0.48
	Harmol	0.999	0.239	0.725	92.0 ± 2.0	2.13 ± 0.80	92.8 ± 4.5	2.28 ± 0.91
	Harmalol	0.999	0.097	0.295	87.5 ± 4.2	5.72 ± 0.33	94.1 ± 3.2	6.56 ± 0.32
	Harmine	0.998	0.111	0.337	93.2 ± 2.1	6.22 ± 0.50	83.6 ± 3.9	6.28 ± 0.44
	Harmaline	0.997	0.422	1.277	89.5 ± 3.5	4.81 ± 0.42	92.4 ± 2.1	5.38 ± 0.48
Cutlassfish	Norharman	0.999	0.094	0.282	95.7 ± 3.2	3.49 ± 0.54	99.9 ± 2.5	3.02 ± 0.39
	Harman	0.999	0.116	0.353	98.4 ± 1.8	3.45 ± 0.43	89.9 ± 2.5	3.02 ± 0.59
	Harmol	0.999	0.458	1.388	92.6 ± 1.6	2.49 ± 0.50	92.4 ± 4.7	2.39 ± 0.44
	Harmalol	0.999	0.052	0.157	88.1 ± 4.3	5.48 ± 0.57	88.4 ± 3.1	4.90 ± 0.50
	Harmine	0.999	0.344	1.042	92.7 ± 2.1	4.94 ± 0.73	85.4 ± 2.8	5.42 ± 0.63
	Harmaline	0.999	0.255	0.773	90.4 ± 3.4	6.65 ± 0.58	89.5 ± 2.6	6.56 ± 0.66
Pork	Norharman	0.999	0.183	0.554	97.7 ± 3.9	3.27 ± 0.42	99.9 ± 3.0	2.90 ± 0.49
	Harman	0.999	0.212	0.644	98.4 ± 2.3	2.95 ± 0.54	90.2 ± 1.7	3.21 ± 0.42
	Harmol	0.999	0.167	0.507	95.7 ± 2.4	5.98 ± 0.92	91.1 ± 4.0	5.53 ± 0.95
	Harmalol	0.999	0.053	0.161	87.2 ± 3.6	4.34 ± 0.34	93.3 ± 2.9	4.38 ± 0.34
	Harmine	0.999	0.074	0.226	93.0 ± 2.4	3.98 ± 0.37	81.4 ± 3.0	4.30 ± 0.31
	Harmaline	0.998	0.164	0.498	91.9 ± 2.9	6.56 ± 0.55	88.8 ± 2.5	6.44 ± 0.51
Beef	Norharman	0.999	0.091	0.275	98.5 ± 4.0	3.36 ± 0.41	99.9 ± 2.9	3.01 ± 0.46
	Harman	0.999	0.087	0.262	98.4 ± 2.1	2.95 ± 0.40	87.3 ± 2.3	2.87 ± 0.56
	Harmol	0.999	0.161	0.487	92.5 ± 2.0	4.89 ± 0.51	96.1 ± 4.4	4.83 ± 0.54
	Harmalol	0.999	0.228	0.692	90.8 ± 3.6	4.90 ± 0.49	89.4 ± 2.8	5.22 ± 0.56
	Harmine	0.999	0.032	0.097	89.9 ± 2.9	5.24 ± 0.47	80.8 ± 3.4	5.40 ± 0.53
	Harmaline	0.999	0.324	0.983	92.4 ± 3.7	2.66 ± 0.64	92.4 ± 2.7	2.35 ± 0.61

R^2 : coefficient of determination; LOD: limit of detection; LOQ: limit of quantitation, RSD: relative standard deviation.

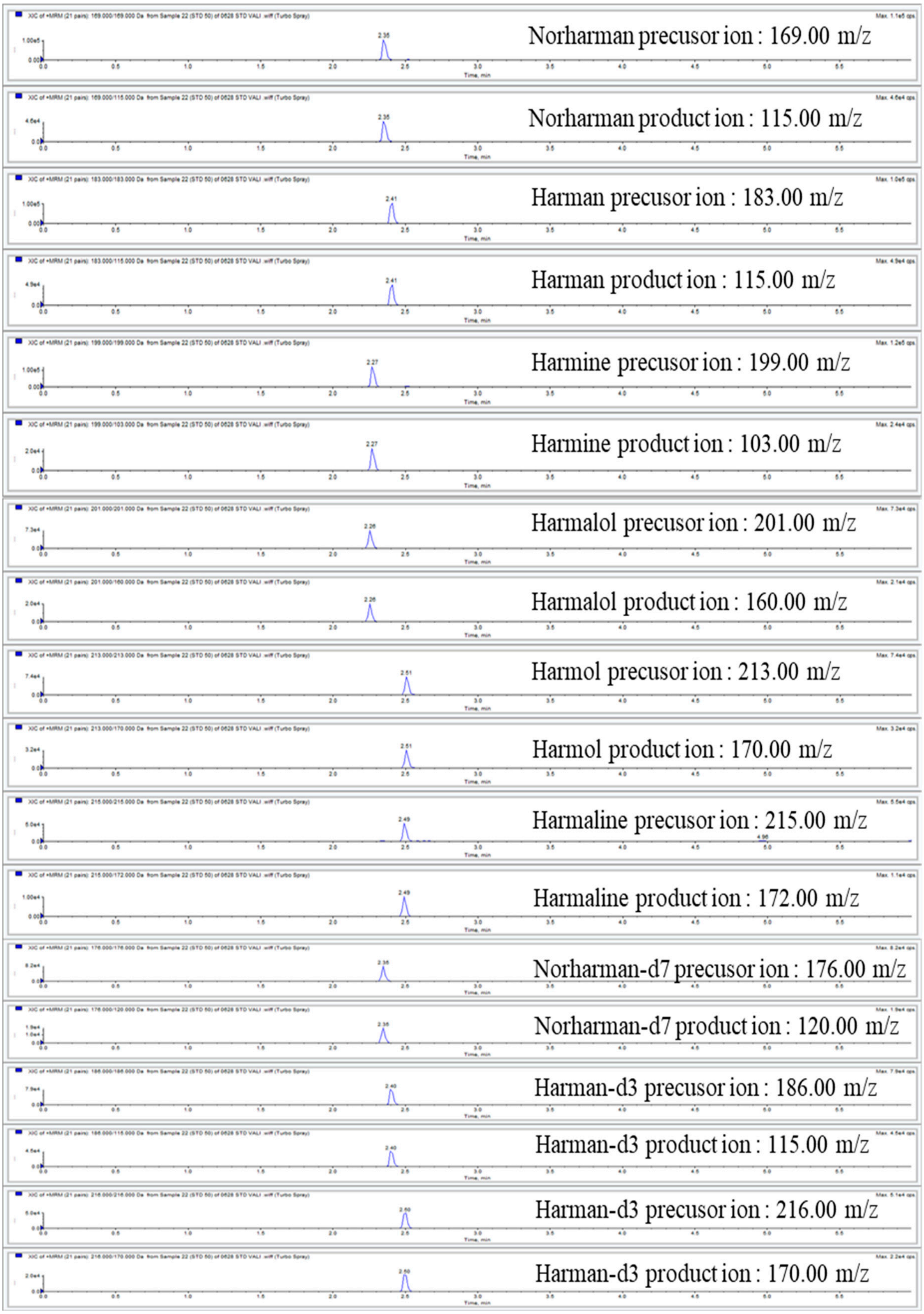

Supplementary Figure S1. LC-MS/MS chromatograms of β-carboline standards and internal standards (50 μg/kg) using selected precursor–product ion transitions.

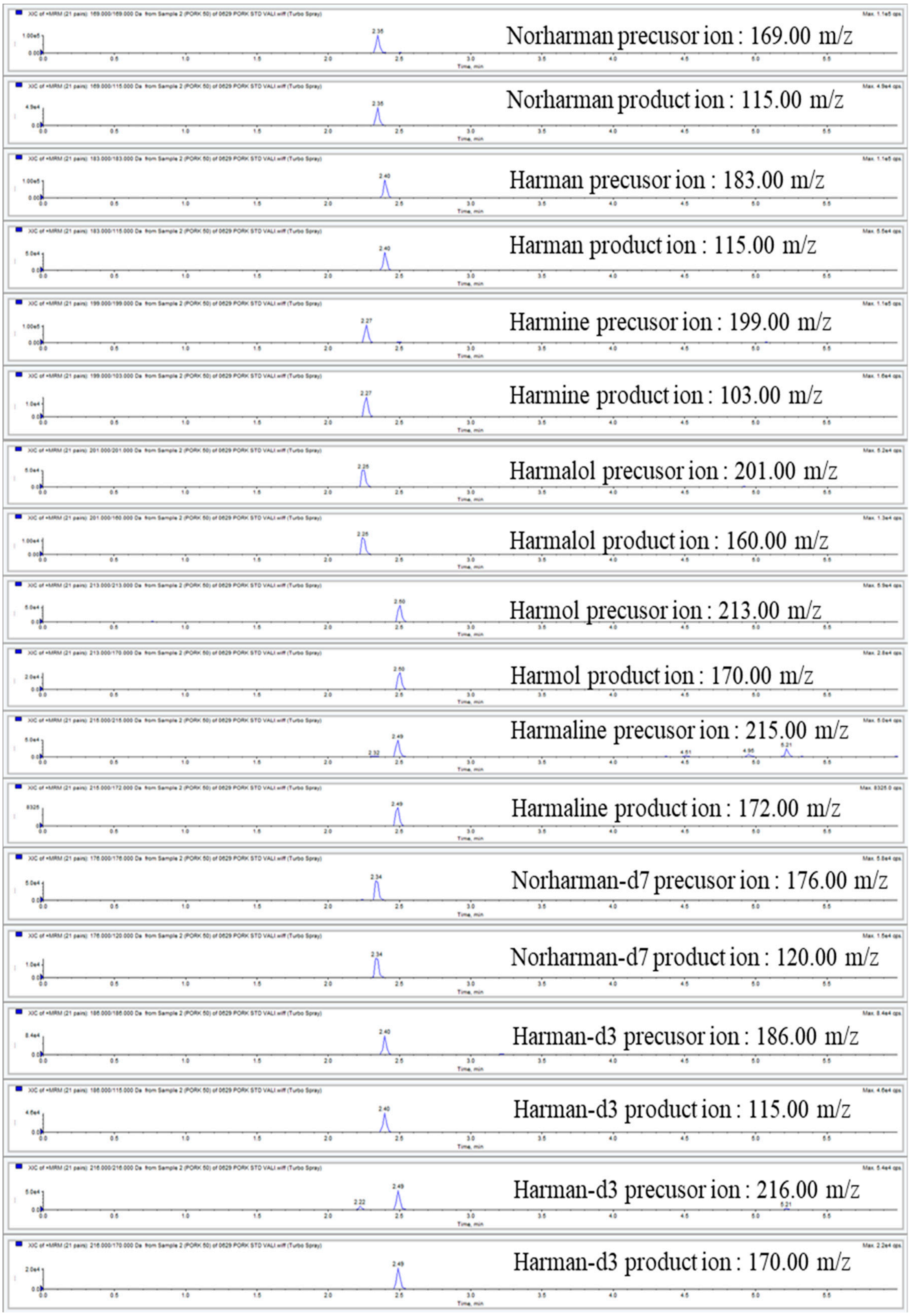

Supplementary Figure S2. LC-MS/MS chromatograms of pork belly spiked with β-carboline standards and internal standard (50 μg/kg)
